# Supplementary material for: Unoccupied aerial system enabled functional modeling of maize height reveals dynamic expression of loci
Source: Plant Direct. 2020 May 10;4(5):e00223. doi: 10.1002/pld3.223 (PMC7212003; doi:10.1002/pld3.223)
Supplement: Supplementary file 1 — Figure S1‐S5 [file PLD3-4-e00223-s001.pdf]

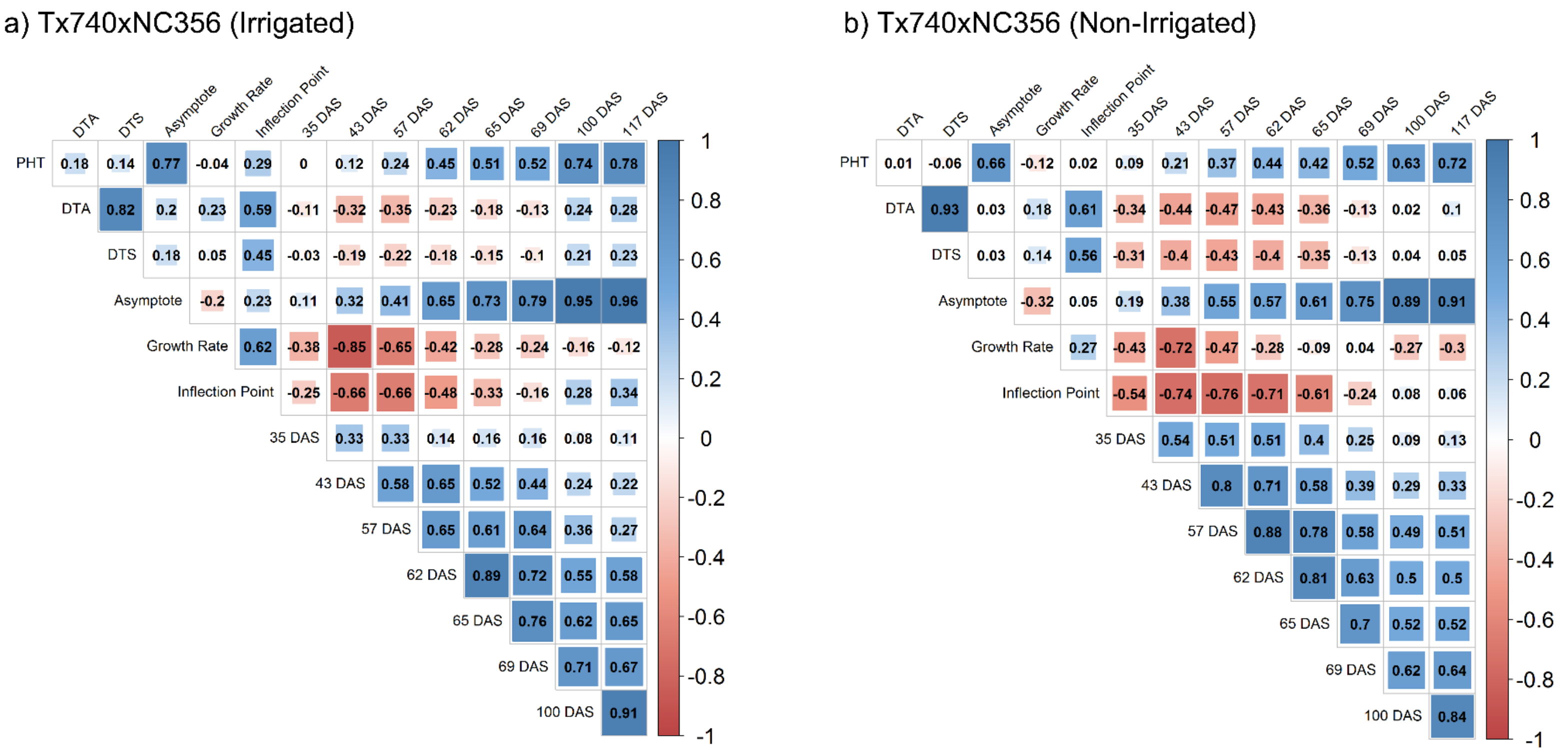

Supplemental Figure S1. Tx740xNC356 correlation heatmaps. Heat map comparing correlations between manual terminal plant height (PHT), flowering time (DTA/DTS), functional parameters (asymptote, growth rate, inflection point), and UAS P95 estimates by flight date for the Tx740xNC356 population under **[a]** irrigated and **[b]** non-irrigated watering regimens. Growth rate is an empirical constant of the Weibull function which defines the maximum absolute growth rate (m d^{-1}).

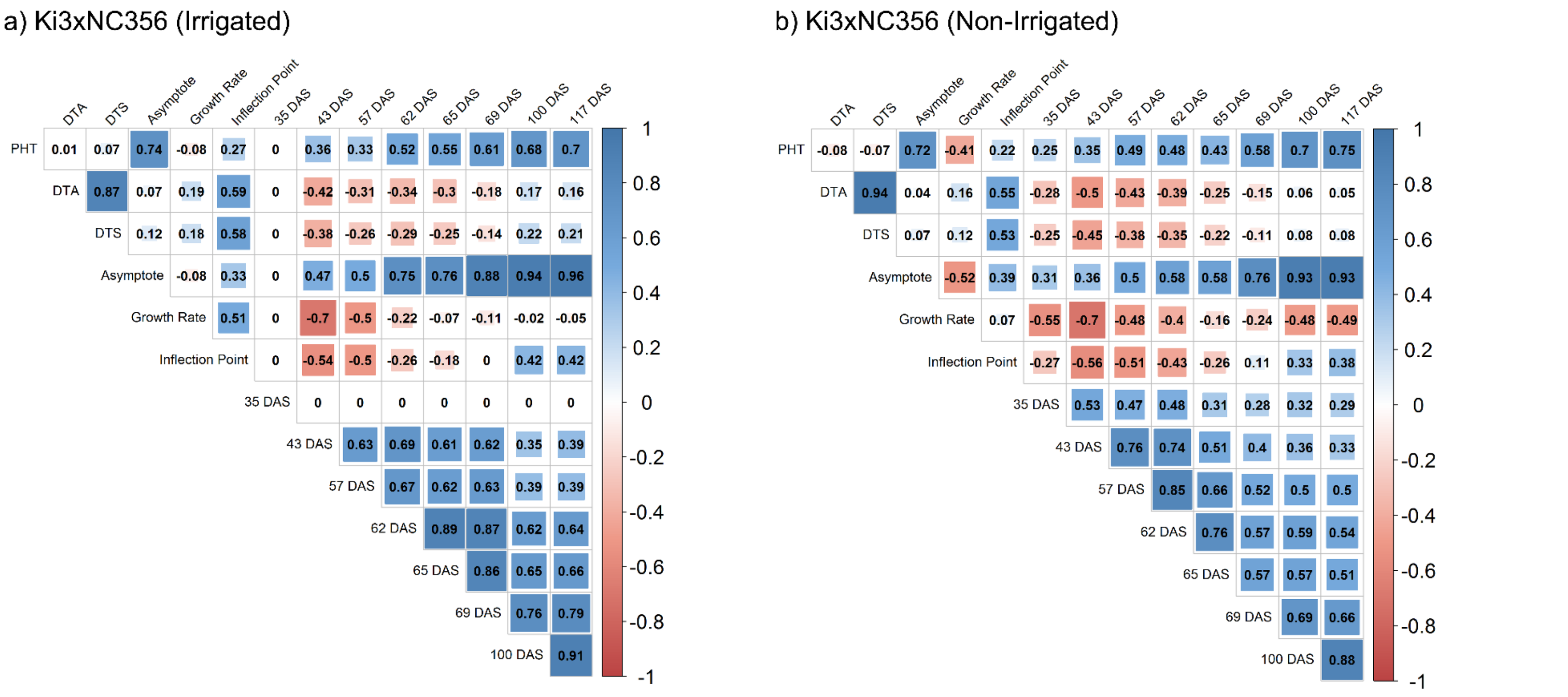

Supplemental Figure S2. Ki3xNC356 correlation heatmaps. Heat map comparing correlations between manual terminal plant height (PHT), flowering time (DTA/DTS), functional parameters (asymptote, growth rate, inflection point), and UAS P95 estimates by flight date for the Ki3xNC356 population under [a] irrigated and [b] non-irrigated watering regimens. Growth rate is an empirical constant of the Weibull function which defines the maximum absolute growth rate (m d-1).

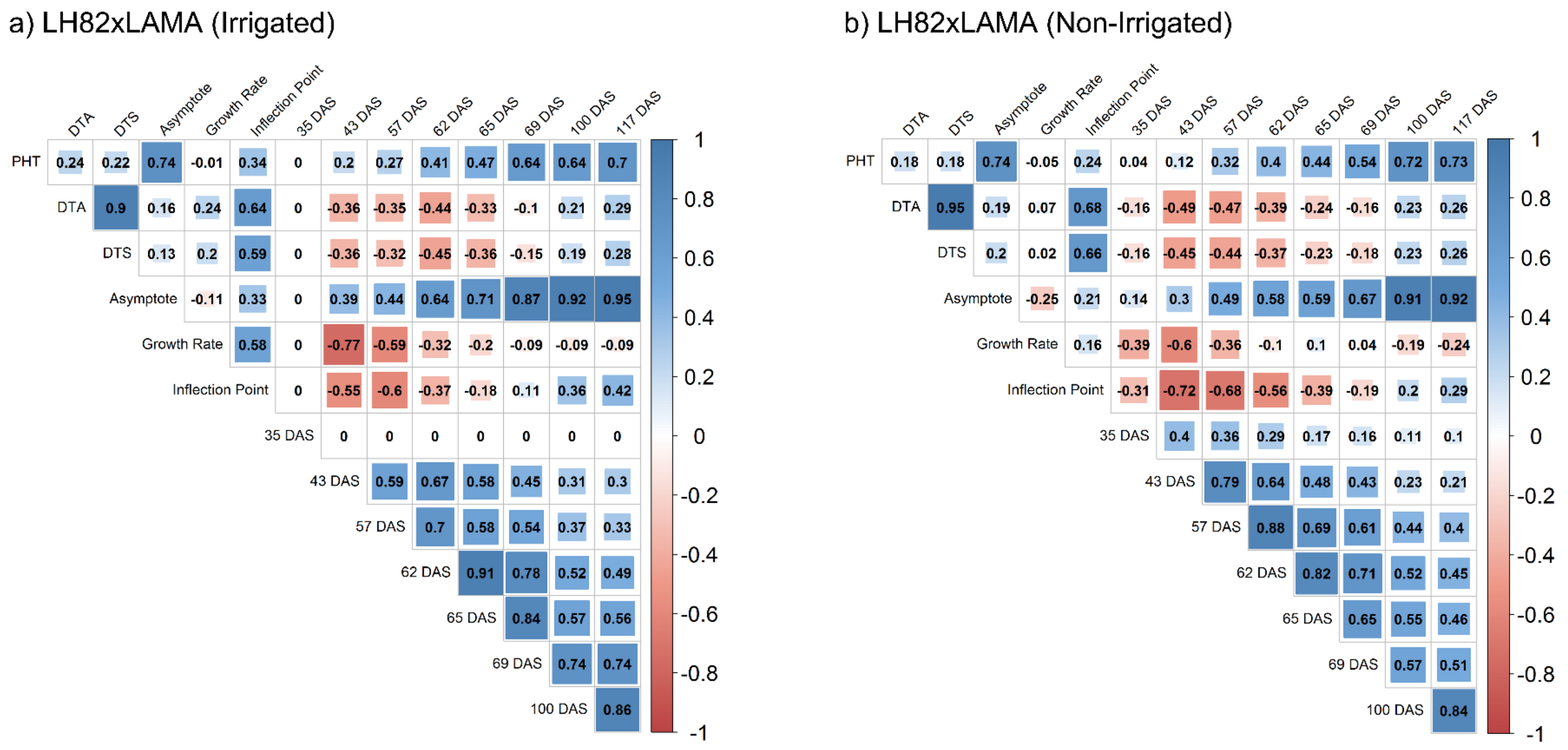

Supplemental Figure S3. LH82xLAMA correlation heatmaps. Heat map comparing correlations between manual terminal plant height (PHT), flowering time (DTA/DTS), functional parameters (asymptote, growth rate, inflection point), and UAS P95 estimates by flight date for the LH82xLAMA population under [a] irrigated and [b] non-irrigated watering regimens. Growth rate is an empirical constant of the Weibull function which defines the maximum absolute growth rate (m d-1).

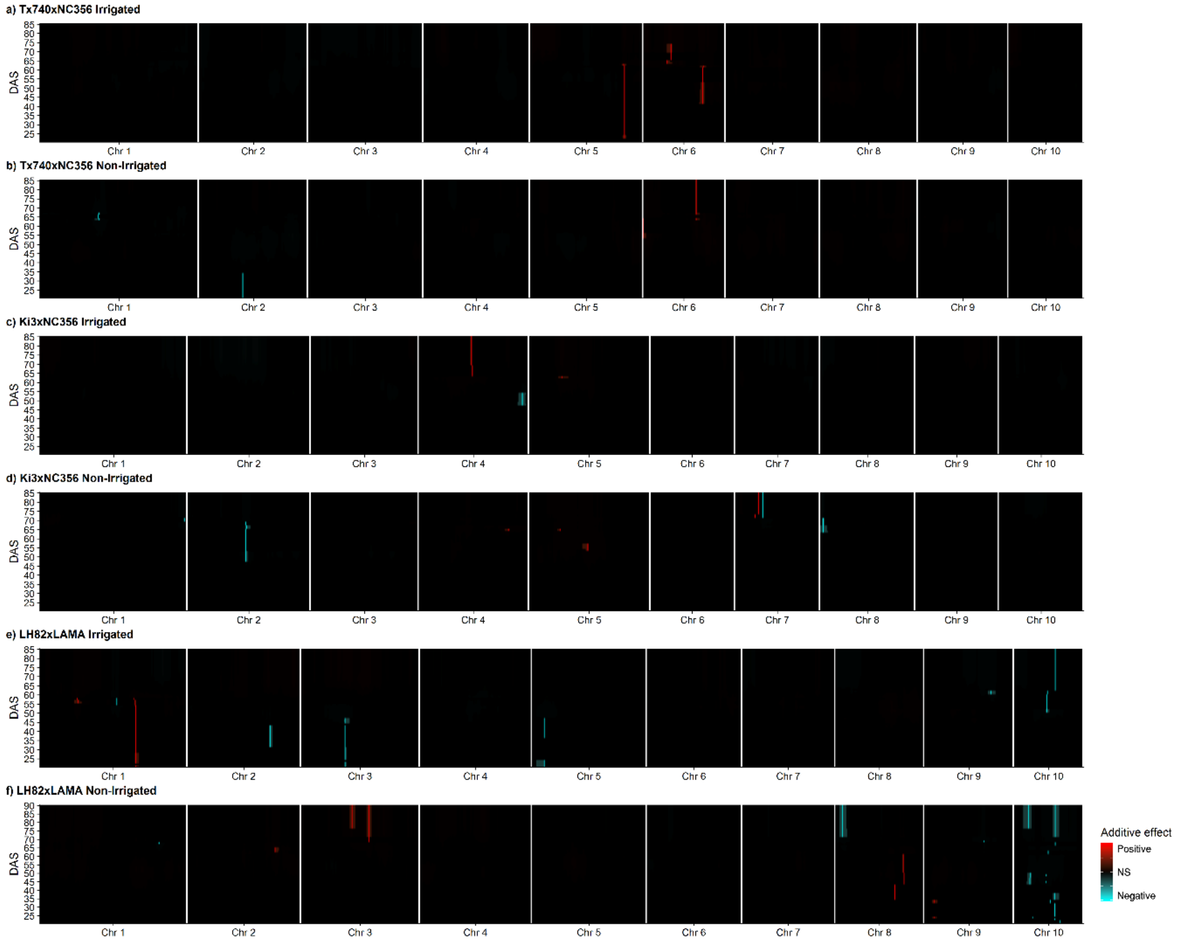

Supplemental Figure S4. Visual representation of temporal QTL. Significant temporal height QTL. Red indicates positive allelic effect estimates, blue indicates negative allelic effect estimates, and black indicate non-significant (NS) genomic regions. Lines represent each of 5316, 5628, and 6231 polymorphic SNPs for the Ki3/NC356, Tx740/NC356, and LH82/LAMA populations, respectively, across the genome (X-axis).

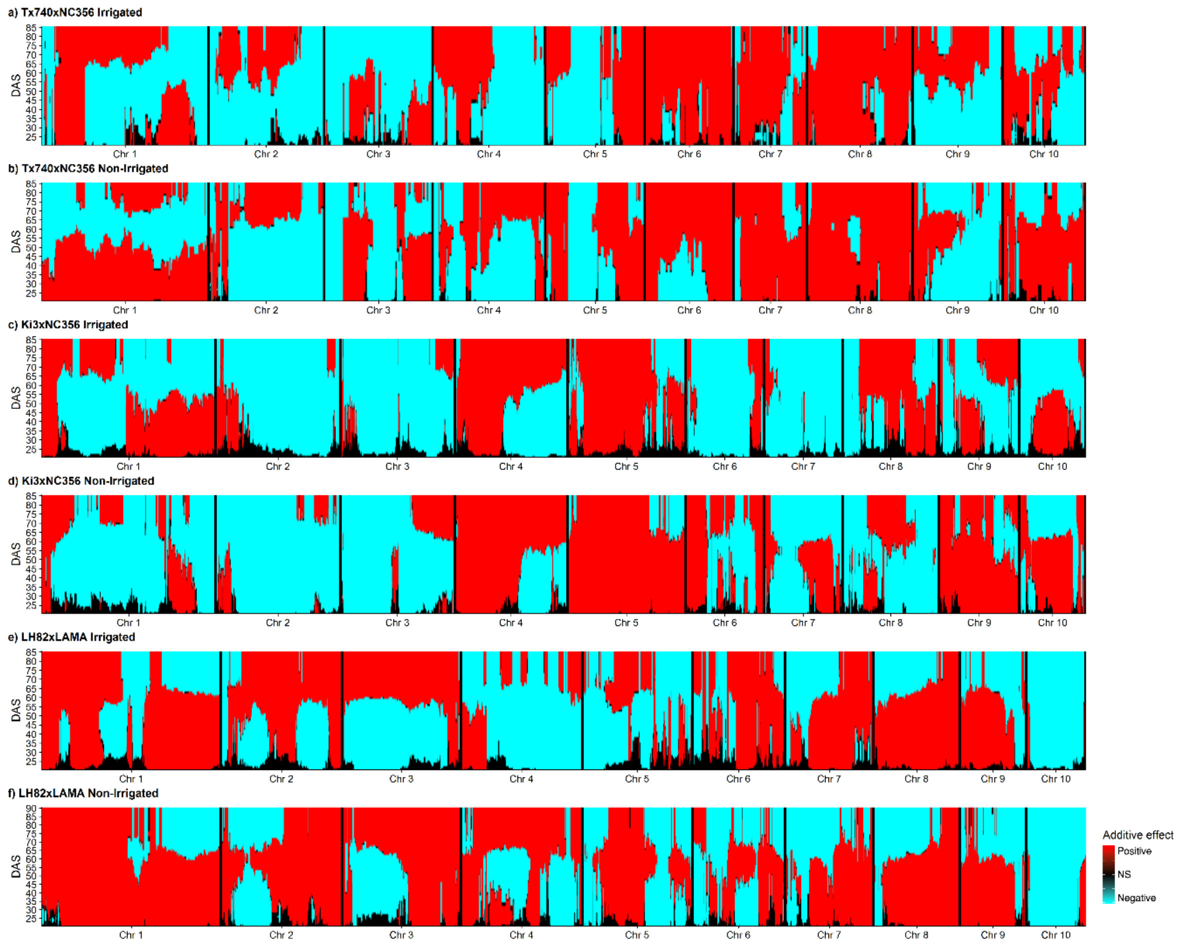

Supplemental Figure S5. Visual representation of temporal allele effect estimate. Visual representation of temporal single marker analysis of the Weibull imputed height estimates. Lines represent each of 5316, 5628, and 6231 polymorphic SNPs for the Ki3/NC356, Tx740/NC356, and LH82/LAMA populations, respectively, across the genome (X-axis).
