# Supplementary material for: Unoccupied aerial system enabled functional modeling of maize height reveals dynamic expression of loci
Source: Plant Direct. 2020 May 10;4(5):e00223. doi: 10.1002/pld3.223 (PMC7212003; doi:10.1002/pld3.223)
Supplement: Supplementary file 2 — Table S1‐S6 [file PLD3-4-e00223-s002.pdf]

Supplemental Table S1. Summary of 2018 UAS flight dates. Summary of 2018 UAS flight dates of the fields containing the Tx740xNC356, Ki3xNC356, and LH82xLAMA populations, including: days after sowing (DAS), the number of images captured, the number of calibrated images, spatial resolution of the mosaic image and mean errors of the GCP geo-referencing.

						GCP Geolocation details				
**Flight date**	**UAS Platform†**	**DAS‡**	**Number Images**	**Calibrated Images**	**Resolution (cm/pix)**	**No. GCP**	**Mean RSME(X) (m)**	**Mean RSME(Y) (m)**	**Mean RSME(Z) (m)**	**Mean RMSE (m)**
3/14/18	RW	0	822	822	1.12	8	0.24	0.25	0.03	0.17
3/30/18,¶	RW	16	505	505	1.04	8	0.13	0.12	0.01	0.08
4/08/18,¶	RW	25	473	473	1.02	8	6.17	2.12	0.08	2.60
4/11/18,¶	RW	28	555	555	1.05	8	0.13	0.11	0.01	0.08
4/18/18,§	RW	35	539	539	1.13	8	0.16	0.15	0.02	0.11
4/26/18,§	RW	43	520	518	1.03	7	0.09	0.09	0.01	0.06
5/10/18,§	RW	57	513	513	1.07	30	0.05	0.05	0.05	0.05
5/15/18,§	RW	62	535	513	1.10	26	5.48	9.41	0.06	4.98
5/18/18,§	RW	65	515	485	1.06	25	0.05	0.06	0.03	0.04
5/22/18,§	RW	69	786	786	0.62	28	0.03	0.03	0.01	0.02
5/25/18,#	RW	72	1484	1315	0.65	30	0.03	0.04	0.03	0.03
5/29/18,¶	RW	76	512	463	1.06	26	0.78	0.91	1.11	0.78
6/01/18,¶	RW	79	521	448	1.04	8	0.01	0.01	<0.01	<0.01
6/05/18,¶	RW	83	-	-	-	-	-	-	-	-
6/07/18,¶	RW	85	-	-	-	-	-	-	-	-
6/13/18,¶	RW	91	513	289	1.04	10	<0.01	<0.01	<0.01	<0.01
6/22/18,¶	RW	100	492	303	1.08	22	0.03	0.02	0.03	0.03
7/03/18,¶	RW	111	290	232	1.10	17	0.19	0.20	0.03	0.14
7/20/18,¶	RW	128	-	-	-	-	-	-	-	-

Supplemental Table S1. Continued.

Flight date	UAS Platform†	DAS‡	Number Images	Calibrated Images	Resolution (cm/pix)	GCP Geolocation details				
						No. GCP	Mean RSME(X) (m)	Mean RSME(Y) (m)	Mean RSME(Z) (m)	Mean RMSE (m)
Tuffwing UAV Mapper										
5/17/18,#	FW	64	67	67	2.70	39	0.02	0.04	0.01	0.04
5/21/18,#	FW	68	66	66	2.68	39	0.02	0.04	0.01	0.04
5/24/18,#	FW	71	65	65	2.59	10	0.01	0.01	<0.01	0.01
6/05/18,#	FW	83	61	61	2.75	10	0.05	0.06	<0.01	0.08
6/08/18,#	FW	86	66	66	2.71	10	0.06	0.06	<0.01	0.08
6/14/18,¶	FW	92	56	56	2.79	10	0.05	0.05	<0.01	0.07
6/18/18,#	FW	96	66	66	2.72	10	0.01	0.02	<0.01	0.03
6/22/18,§	FW	100	68	68	2.72	10	0.05	0.06	<0.01	0.08
7/09/18,§	FW	117	67	67	2.77	10	0.01	0.01	0.01	0.02
7/16/18,#	FW	124	-	-	-	-	-	-	-	-
7/25/18,#	FW	133	-	-	-	-	-	-	-	-

† FW: Fixed wing; RW: Rotary wing.
‡ DAS: Days after sowing.
§ Final set of high quality flights.
¶ Qualitatively discarded due to black holes or lack of structural data.
# Dataset discarded due to statistical deviation from growth trajectory theory.

Supplemental Table S2. Descriptive statistics of UAS flight dates by population. Summary statistics of the entries for each population (Tx740xNC356, Ki3xNC356, and LH82xLAMA) across the six identified flight dates with high quality point clouds for the irrigated and non-irrigated trials.

		Non-Irrigated Trial							Irrigated Trial						
Population	DAS†	N Genotypes	Mean	Std. Dev.	Min	Max	Female Mean	Male Mean	N	Mean	Std. Dev.	Min	Max	Female Mean	Male Mean
Tx740xNC356	35	101	0.08	0.02	0.04	0.12	0.08	0.11	87	0.07	0.01	0.06	0.11	0.05	0.11
Tx740xNC356	43	101	0.19	0.05	0.09	0.32	0.17	0.24	100	0.17	0.04	0.08	0.27	0.11	0.21
Tx740xNC356	57	101	0.65	0.06	0.48	0.84	0.63	0.69	100	0.52	0.05	0.39	0.64	0.49	0.59
Tx740xNC356	62	101	0.88	0.06	0.65	1.07	0.88	0.90	100	0.85	0.07	0.63	1.03	0.82	0.88
Tx740xNC356	65	101	1.05	0.07	0.70	1.26	1.07	1.08	100	1.00	0.07	0.80	1.22	0.96	1.02
Tx740xNC356	69	101	1.03	0.07	0.83	1.20	1.05	0.95	100	1.06	0.07	0.79	1.24	1.01	1.10
Tx740xNC356	100	101	1.12	0.08	0.91	1.34	1.17	1.17	100	1.07	0.09	0.88	1.39	1.06	1.11
Tx740xNC356	117	101	1.10	0.07	0.91	1.28	1.13	1.08	100	1.04	0.08	0.86	1.34	1.05	1.06
Ki3xNC356	35	237	0.07	0.02	0.04	0.12	0.05	0.11	196	0.01	0.00	0.01	0.01	0.01	0.01
Ki3xNC356	43	237	0.19	0.04	0.09	0.33	0.17	0.24	238	0.16	0.04	0.06	0.28	0.17	0.17
Ki3xNC356	57	237	0.66	0.06	0.46	0.91	0.64	0.68	238	0.55	0.05	0.36	0.69	0.54	0.51
Ki3xNC356	62	237	0.86	0.07	0.58	1.10	0.87	0.88	238	0.88	0.08	0.67	1.21	0.82	0.88
Ki3xNC356	65	237	1.02	0.06	0.77	1.17	1.03	1.05	238	1.03	0.08	0.68	1.32	0.97	1.00
Ki3xNC356	69	237	1.00	0.09	0.72	1.24	1.00	1.04	238	1.08	0.10	0.70	1.48	1.02	1.10
Ki3xNC356	100	237	1.07	0.08	0.81	1.36	1.05	1.17	238	1.04	0.10	0.80	1.32	1.02	1.11
Ki3xNC356	117	237	1.02	0.09	0.82	1.32	1.02	1.07	238	1.01	0.09	0.79	1.30	0.98	1.06

Supplemental Table S2. Continued.

Population	DAS†	Non-Irrigated Trial							Irrigated Trial						
		N	Mean	Std. Dev.	Min	Max	Female Mean	Male Mean	N	Mean	Std. Dev.	Min	Max	Female Mean	Male Mean
LH82xLAMA	35	174	0.08	0.01	0.06	0.11	0.06	0.06	101	0.00	0.00	0.00	0.00	0.00	0.00
LH82xLAMA	43	174	0.19	0.04	0.08	0.33	0.24	0.09	175	0.15	0.04	0.05	0.29	0.18	0.07
LH82xLAMA	57	174	0.68	0.06	0.45	0.88	0.72	0.58	175	0.58	0.05	0.42	0.74	0.61	0.45
LH82xLAMA	62	174	0.90	0.09	0.59	1.15	0.91	0.78	175	0.92	0.08	0.73	1.18	0.95	0.81
LH82xLAMA	65	174	1.08	0.07	0.71	1.25	1.05	1.07	175	1.08	0.08	0.77	1.30	1.05	1.00
LH82xLAMA	69	174	1.04	0.07	0.87	1.25	0.84	0.89	175	1.13	0.10	0.92	1.39	1.02	1.08
LH82xLAMA	100	174	1.10	0.08	0.85	1.37	0.92	1.16	175	1.04	0.09	0.86	1.28	0.89	1.09
LH82xLAMA	117	174	1.04	0.08	0.83	1.32	0.84	1.14	175	0.99	0.10	0.79	1.24	0.81	1.06

† DAS: Days after sowing.

Supplemental Table S3. Manual plant height QTL. Summary of QTL identified using manual terminal plant height as the associated phenotype. Physical locations (bp) based on B73 RefGen_3, AGPv3.

Population	Trt†	Chromosome	Position (cM)	LOD§	PVE‡	Add (m)¶	Left Marker Position (cM)	Right Marker Position (cM)	Left Marker Position (bp)	Right Marker Position (bp)
Ki3xNC356	D	2	69	4.3	8.2	-0.03	68.5	69.5	98,935,095	101,781,010
Ki3xNC356	D	8	12	3.0	5.6	-0.03	10.5	13.5	5,024,449	5,912,287
Ki3xNC356	I	2	70	3.0	5.8	-0.03	69.5	70.5	113,899,778	127,959,743
Ki3xNC356	I	6	62	2.6	5.1	-0.03	61.5	62.5	148,942,282	149,680,023
Ki3xNC356	I	8	14	2.9	5.5	-0.03	13.5	15.5	6,810,510	7,748,559
LH82xLAMA	D	3	86	2.8	6.4	0.03	85.5	86.5	169,071,582	169,218,337
LH82xLAMA	I	1	172	2.8	6.4	-0.03	171.5	172.5	280,342,748	280,578,622
LH82xLAMA	I	8	65	2.2	6	-0.03	61.5	66.5	125,523,343	128,428,318
Tx740xNC356	D	1	176	2.0	9.4	-0.04	171.5	178.5	281,709,021	283,430,713

† Trt: Irrigation regimen; irrigated (I) and non-irrigated (D).
§ LOD: Logarithm of odds calculated as LOD = $-\log_{10}$(p-value).
‡ PVE: Prevent variation explained.
¶ Add: Estimated additive effect size of the QTL.

Supplemental Table S4. Flowering time QTL Summary of significant QTL for flowering time. Physical locations (bp) based on B73 RefGen_3, AGPv3.

Population	Trt†	Trait‡	Chr§	Position (cM)	Left Marker	Right Marker	LOD¶	PVE#	Add†† (d)	Left CI‡‡ (bp)	Right CI‡‡ (bp)
Ki3xNC356	I	DTA	2	24	AgR_02478	AgR_04085	6.77	5.08	0.63	10603658	11134513
Ki3xNC356	I	DTA	2	30	AgR_00649	AgR_00650	15.01	12.09	-0.98	13342033	13342154
Ki3xNC356	D	DTA	2	34	AgR_14537	AgR_10838	2.09	4.05	-0.42	15125786	15246819
Ki3xNC356	I	DTS	2	35	AgR_02349	AgR_14540	5.58	10.68	-0.76	15580072	15979202
Ki3xNC356	D	DTS	6	24	AgR_05809	AgR_16068	2.02	4.10	-0.49	89403767	89687544
Ki3xNC356	I	DTS	6	87	AgR_07216	AgR_05955	2.17	3.98	0.46	162645223	163665243
Ki3xNC356	D	DTA	9	60	AgR_01594	AgR_16910	2.07	4.03	0.42	133886762	133897924
LH82xLAMA	I	DTS	1	129	AgR_03912	AgR_01056	2.03	3.91	-0.49	223165736	224078702
LH82xLAMA	D	DTA	1	168	AgR_00790	AgR_00109	4.46	10.11	-0.62	275833355	277585472
LH82xLAMA	I	DTA	1	169	AgR_10766	AgR_10769	7.42	15.25	-0.73	278195980	278603093
LH82xLAMA	D	DTS	1	181	AgR_00413	AgR_00912	3.75	7.01	-0.56	287290866	287291452
LH82xLAMA	I	DTS	1	182	AgR_17266	AgR_07760	5.43	11.64	-0.84	287335033	287586834
LH82xLAMA	D	DTS	2	28	AgR_00736	AgR_10834	3.94	7.22	-0.57	11299939	11946310
LH82xLAMA	I	DTA	3	68	AgR_15093	AgR_02600	2.69	5.22	0.43	154076377	154660873
LH82xLAMA	D	DTS	3	68	AgR_15093	AgR_02600	2.52	4.53	0.46	154076377	154660873
LH82xLAMA	I	DTS	8	62	AgR_09615	AgR_13174	6.11	13.27	-0.90	120610321	122427978
LH82xLAMA	D	DTS	8	62	AgR_09615	AgR_13174	8.58	16.71	-0.87	120610321	122427978
LH82xLAMA	I	DTA	9	36	AgR_06613	AgR_06616	2.69	5.23	-0.44	15307137	15409063
LH82xLAMA	D	DTA	9	74	AgR_06777	AgR_01799	3.11	6.93	-0.52	110916495	130918887
LH82xLAMA	I	DTS	9	103	AgR_13529	AgR_16949	3.78	7.94	-0.70	149276973	149426980
LH82xLAMA	I	DTA	9	104	AgR_16951	AgR_09895	2.70	5.24	-0.43	149747320	149897954
LH82xLAMA	D	DTS	9	106	AgR_09895	AgR_09897	3.99	7.26	-0.57	149897954	149971831
Tx740xNC356	I	DTA	8	60	AgR_09612	AgR_06423	2.07	9.45	0.55	117761454	118224127

Supplemental Table S4. Continued.

Population	Trt†	Trait‡	Chr§	Position (cM)	Left Marker	Right Marker	LOD¶	PVE#	Add†† (d)	Left CI‡‡ (bp)	Right CI‡‡ (bp)
Tx740xNC356	D	DTS	10	85	AgR_07121	AgR_13974	2.28	11.23	-0.65	144462556	145465473
Tx740xNC356	I	DTS	10	89	AgR_13777	AgR_17166	2.94	14.03	-0.72	145795322	145934793

† Trt: Treatment; I: Irrigated; D: Non-Irrigated.
‡ DTA: Days to anthesis; DTS: Days to silking.
§ Chr: Chromosome.
¶ LOD: $-\log_{10}$(p-value).
# PVE: Percent variation explained.
†† Additive effect estimate.
‡‡ CI: Confidence interval. Physical location based on based on B73 RefGen_3, AGPv3.

Supplemental Table S5. Function growth parameter QTL. Summary of significant QTL for functional parameters of the Weibull sigmoid function. Physical locations (bp) based on B73 RefGen_3, AGPv3. Growth rate (GR) is an empirical constant of the Weibull function which defines the maximum absolute growth rate (m d^{-1}).

Population	Trt†	Trait‡	Chr§	Position (cM)	LOD¶	PVE#	Add††	Left marker (bp)	Right marker (bp)	Closest candidate gene	Gene function	Distance from gene (bp)
Ki3xNC356	I	Asym	2	59	2.46	4.45	-0.02	48405262	48664915	GRMZM2G017187	auxin response factor 1; auxin response factor 9, putative, expressed	1026060
Ki3xNC356	D	IP	3	94	3.57	6.27	0.36	212692256	212698619	GRMZM2G126260	auxin efflux carrier family protein; auxin efflux carrier component, putative, expressed	2206613
Ki3xNC356	I	Asym	4	61	4.92	9.23	0.03	156384593	156517564	GRMZM2G134023	brassinosteroid-responsive RING-H2; zinc finger, C3HC4 type domain containing protein, expressed	259336.5
Ki3xNC356	I	IP	4	61	2.21	4.28	0.22	156384593	156517564	GRMZM2G134023	brassinosteroid-responsive RING-H2; zinc finger, C3HC4 type domain containing protein, expressed	259336.5
Ki3xNC356	I	IP	4	119	2.66	5.39	0.25	237391404	237610499	AC196708.3_FG006	SAUR-like auxin-responsive protein family ; OsSAUR5 - Auxin-responsive SAUR gene family member, expressed	187124.5
Ki3xNC356	D	IP	4	119	3.12	5.41	0.33	237391404	237610499	AC196708.3_FG006	SAUR-like auxin-responsive protein family ; OsSAUR5 - Auxin-responsive SAUR gene family member, expressed	187124.5
Ki3xNC356	I	Asym	5	38	2.28	3.93	0.02	21893133	21893252	GRMZM2G113135	SAUR-like auxin-responsive protein family ; CPuORF40 - conserved peptide uORF-containing transcript, expressed	1105270
Ki3xNC356	D	Asym	7	27	13.04	7.35	0.04	25781951	26153406	GRMZM2G075715	auxin response factor 6; auxin response factor, putative, expressed	13766771
Ki3xNC356	D	Asym	7	32	21.45	13.37	-0.05	95447274	96847624	GRMZM2G391795	alpha/beta-Hydrolases superfamily protein; gibberellin receptor GID1L2, putative, expressed	11778171
Ki3xNC356	D	IP	8	19	2.65	4.63	-0.30	8731594	9875920	GRMZM2G000489	alpha/beta-Hydrolases superfamily protein; gibberellin receptor GID1L2, putative, expressed	5630336
Ki3xNC356	D	IP	10	41	3.37	5.90	-0.34	84071836	86777777	GRMZM2G072632	Auxin efflux carrier family protein; auxin efflux carrier component, putative, expressed	4860010
LH82xLAMA	I	GR	1	135	4.03	7.36	-0.27	229187847	229850714	GRMZM2G153233	auxin response factor 16; auxin response factor 18, putative, expressed	1200114

Supplemental Table S5. Continued.

Population	Trt†	Trait‡	Chr§	Position (cM)	LOD¶	PVE#	Add††	Left marker (bp)	Right marker (bp)	Closest candidate gene	Gene function	Distance from gene (bp)
LH82xLAMA	D	IP	1	145	3.23	7.65	-0.39	251713072	252736144	GRMZM2G341460	2-oxoglutarate (2OG) and Fe(II)-dependent oxygenase superfamily protein; gibberellin 20 oxidase 2, putative, expressed	2399240
LH82xLAMA	I	Asym	1	173	2.04	5.35	-0.02	280634097	281590394	vp8	viviparous8; small plant	5211076
LH82xLAMA	I	IP	1	176	4.31	11.58	-0.41	283600596	284011134	vp8	viviparous8; small plant	2517456
LH82xLAMA	I	GR	2	117	4.78	8.77	0.29	212246084	212544633	GRMZM2G064941	Auxin efflux carrier family protein; auxin efflux carrier component, putative, expressed	254540.5
LH82xLAMA	I	GR	3	62	3.62	6.56	0.25	140845175	142249525	GRMZM2G116204	endoplasmic reticulum auxin binding protein 1; auxin-binding protein 4 precursor, putative, expressed	7656087
LH82xLAMA	I	IP	3	67	2.66	6.98	0.32	151971786	154075136	GRMZM2G338259	auxin response factor 2; auxin response factor, putative, expressed	3461005
LH82xLAMA	D	IP	3	69	3.12	7.78	0.39	154668842	155959968	GRMZM2G338259	auxin response factor 2; auxin response factor, putative, expressed	1170061
LH82xLAMA	D	Asym	3	72	2.08	3.37	0.02	158668137	159817878	sdw2	short plant	830009.5
LH82xLAMA	D	Asym	3	96	2.14	3.42	0.02	176562816	177536662	na1	dwarf plant	1942864
LH82xLAMA	I	GR	5	28	3.55	6.41	0.25	7996372	8175228	GRMZM2G130675	SAUR-like auxin-responsive protein family ; OsSAUR1 - Auxin-responsive SAUR gene family member, expressed	2835967
LH82xLAMA	D	Asym	8	10	2.26	3.86	-0.02	5024449	5024479	GRMZM2G000489	alpha/beta-Hydrolases superfamily protein; gibberellin receptor GID1L2, putative, expressed	9909629
LH82xLAMA	D	IP	8	81	5.38	13.02	-0.51	151312347	151664188	clt1	dwarf plant	1752094
LH82xLAMA	I	GR	8	117	3.08	5.60	-0.23	172351557	172463767	GRMZM2G031724	Arabidopsis thaliana gibberellin 2-oxidase 1; gibberellin 2-beta-dioxygenase, putative, expressed	1441662
LH82xLAMA	D	GR	9	14	2.45	6.87	-0.20	5289590	6124392	GRMZM2G307440	alpha/beta-Hydrolases superfamily protein; gibberellin receptor GID1L2, putative, expressed	873137
LH82xLAMA	D	Asym	10	20	2.09	3.48	-0.02	5874629	6537612	cr4	Crinkly4; short plant	616213.5
LH82xLAMA	D	GR	10	20	2.32	6.20	0.20	5874629	6537612	cr4	Crinkly4; short plant	616213.5
LH82xLAMA	I	Asym	10	58	4.11	12.23	-0.03	136083608	136247247	GRMZM2G397684	brassinosteroid-responsive RING-H2; zinc finger, C3HC4 type domain containing protein, expressed	158067.5

Supplemental Table S5. Continued.

Population	Trt†	Trait‡	Chr§	Position (cM)	LOD¶	PVE#	Add††	Left marker (bp)	Right marker (bp)	Closest candidate gene	Gene function	Distance from gene (bp)
LH82xLAMA	D	Asym	10	58	2.19	3.59	-0.02	136083608	136247247	GRMZM2G397684	brassinosteroid-responsive RING-H2; zinc finger, C3HC4 type domain containing protein, expressed	158067.5
Tx740xNC356	I	GR	1	167	2.19	9.24	-0.23	273833874	274877616	GRMZM5G899865	SAUR-like auxin-responsive protein family ; OsSAUR24 - Auxin-responsive SAUR gene family member, expressed	6791261
Tx740xNC356	D	IP	2	87	2.35	10.64	0.40	182727527	183505515	GRMZM2G045243	SAUR-like auxin-responsive protein family ; OsSAUR37 - Auxin-responsive SAUR gene family member, expressed	502507
Tx740xNC356	I	GR	5	119	3.01	15.02	-0.30	205748909	206263616	GRMZM2G074267	Auxin efflux carrier family protein; auxin efflux carrier component, putative, expressed	722594.5
Tx740xNC356	D	Asym	6	67	3.20	14.31	0.02	141909772	143997309	dwil1	dwarf & irregular leaf1	346506.5

† Trt: Treatment; I: Irrigated; D: Non-Irrigated.
‡ Asym: Asymptote; IP: Inflection Point; GR: Growth Rate.
§ Chr: Chromosome.
¶ LOD: $-\log_{10}$(p-value).
# PVE: Percent variation explained.
†† Additive effect estimate; Asym (m); IP (DAS); GR (DAS^{-1}).

Supplemental Table S6. Temporal height QTL. Summary of significant temporal QTL for height estimates imputed from Weibull sigmoid curve at discrete time points (i.e. DAS where significant associations were identified.). Physical locations (bp) based on B73 RefGen_3, AGPv3.

Population	Trt †	Chr ‡	Pos (cM)	Sig. DAS§	Peak LOD¶ DAS††	LOD ¶	PVE #	Add (m) ††	Left marker (bp)	Right marker (bp)	Closest candidate gene	Gene function	Distance from gene (bp)
LH82xLAMA	I	1	52	57-58	58	2.77	5.96	0.02	38250642	38633093	GRMZM2G331638	Auxin-responsive family protein; auxin-responsive protein-related, putative, expressed	13698348
LH82xLAMA	I	1	53	56-57	57	2.18	4.76	0.02	39742242	40356668	GRMZM2G423851	O-fucosyltransferase family protein; auxin-independent growth promoter protein, putative, expressed	13899169
Tx740xNC356	D	1	73	65-67	65	2.41	10.86	-0.02	79236413	80698058	AC204821.3_FG004	auxin response factor 10; indole-3-acetate beta-glucosyltransferase, putative, expressed	12058770
Tx740xNC356	D	1	74	64-67	67	2.32	10.43	-0.02	81440994	81549504	AC204821.3_FG004	auxin response factor 10; indole-3-acetate beta-glucosyltransferase, putative, expressed	10530756
LH82xLAMA	I	1	108	55-58	58	4.31	9.79	-0.02	198779072	198817809	GRMZM2G031065	alpha/beta-Hydrolases superfamily protein; gibberellin receptor GID1L2, putative, expressed	943009
LH82xLAMA	I	1	132	26-59	58	5.60	12.54	0.02	225044054	226136399	GRMZM2G414727	SAUR-like auxin-responsive protein family ; CPuORF40 - conserved peptide uORF-containing transcript, expressed	1217180
LH82xLAMA	I	1	134	20-59	55	5.38	13.59	0.03	229073049	229187847	GRMZM2G382393	Auxin efflux carrier family protein; auxin efflux carrier component, putative, expressed	1317326
LH82xLAMA	I	1	135	20-57	55	5.22	13.19	0.03	229187847	229850714	GRMZM2G153233	auxin response factor 16; auxin response factor 18, putative, expressed	1200114
LH82xLAMA	D	1	168	68	68	2.02	5.18	-0.02	275833355	277585472	GRMZM5G899865	SAUR-like auxin-responsive protein family ; OsSAUR24 - Auxin-responsive SAUR gene family member, expressed	0
Ki3xNC356	D	1	167	70-73	71	2.49	4.49	-0.02	298277113	298497315	AC203966.5_FG005	gibberellin 20 oxidase 2; gibberellin 20 oxidase 1, putative, expressed	1141378
Tx740xNC356	D	2	55	20-42	21	2.73	12.40	0.00	38192289	41023316	GRMZM2G121700	2-oxoglutarate (2OG) and Fe(II)-dependent oxygenase superfamily protein; gibberellin 20 oxidase 2, putative, expressed	1414534
Ki3xNC356	D	2	67	48-69	61	2.59	5.32	-0.01	80271334	80586731	GRMZM5G848945	auxin signaling F-box 3; OsFBL16 - F-box domain and LRR containing protein, expressed	15391130
Ki3xNC356	D	2	68	51-67	66	2.29	4.65	-0.01	83187294	87042364	GRMZM2G451037	SAUR-like auxin-responsive protein family ; OsSAUR4 - Auxin-responsive SAUR gene family member, expressed	19614474
LH82xLAMA	I	2	117	32-43	37	2.28	5.14	0.00	212246084	212544633	GRMZM2G064941	Auxin efflux carrier family protein; auxin efflux carrier component, putative, expressed	254541
LH82xLAMA	D	2	124	63-65	64	2.16	5.96	0.02	217637431	219258619	GRMZM2G062019	carboxyesterase 18; gibberellin receptor GID1L2, putative, expressed	708484
LH82xLAMA	I	3	62	20-47	37	2.59	6.30	0.00	140845175	142249525	GRMZM2G116204	endoplasmic reticulum auxin binding protein 1; auxin-binding protein 4 precursor, putative, expressed	7656087
LH82xLAMA	D	3	72	77-85	85	2.08	5.31	0.02	158668137	159817878	sdw2	short plant	830010
LH82xLAMA	D	3	96	69-85	70	2.18	5.53	0.02	176562816	177536662	na1	dwarf plant	1942864

Supplemental Table S6. Continued.

Population	Trt †	Chr ‡	Pos (cM)	Sig. DAS§	Peak LOD¶ DAS††	LOD ¶	PVE #	Add (m) ††	Left marker (bp)	Right marker (bp)	Closest candidate gene	Gene function	Distance from gene (bp)
Ki3xNC356	I	4	61	63-85	82	5.03	10.29	0.03	156384593	156517564	GRMZM2G134023	brassinosteroid-responsive RING-H2; zinc finger, C3HC4 type domain containing protein, expressed	259337
Ki3xNC356	I	4	62	63-69	69	4.13	8.45	0.02	156998152	157879556	GRMZM2G134023	brassinosteroid-responsive RING-H2; zinc finger, C3HC4 type domain containing protein, expressed	1247112
Ki3xNC356	D	4	104	65-67	67	2.23	4.54	0.01	218940880	219145633	GRMZM2G326114	2-oxoglutarate (2OG) and Fe(II)-dependent oxygenase superfamily protein; gibberellin 20 oxidase 2, putative, expressed	15224555
Ki3xNC356	I	4	120	48-54	52	2.11	4.40	-0.01	237391404	237610499	AC196708.3_FG006	SAUR-like auxin-responsive protein family ; OsSAUR5 - Auxin-responsive SAUR gene family member, expressed	187125
LH82xLAMA	I	5	17	21-52	39	2.65	6.48	-0.01	5105202	5211414	GRMZM2G130675	SAUR-like auxin-responsive protein family ; OsSAUR1 - Auxin-responsive SAUR gene family member, expressed	91525
Ki3xNC356	D	5	36	63-65	64	2.11	4.06	0.01	17344330	18452570	GRMZM2G060940	2-oxoglutarate (2OG) and Fe(II)-dependent oxygenase superfamily protein; gibberellin 20 oxidase 2, putative, expressed	273100
Ki3xNC356	I	5	38	63	63	2.08	4.33	0.01	21893133	21893252	GRMZM2G113135	SAUR-like auxin-responsive protein family ; CPuORF40 - conserved peptide uORF-containing transcript, expressed	1105270
Ki3xNC356	D	5	68	54-57	56	2.02	3.95	0.01	170416302	171276656	GRMZM2G702026	auxin response factor 1; auxin response factor 7, putative, expressed	2961382
Tx740xNC356	I	5	118	24-63	63	2.19	9.94	0.02	204585691	205239681	GRMZM5G885274	GRAS family transcription factor; gibberellin response modulator protein, putative, expressed	171258
Tx740xNC356	I	5	119	23-63	56	4.90	20.89	0.03	205748909	206263616	GRMZM2G074267	Auxin efflux carrier family protein; auxin efflux carrier component, putative, expressed	722595
Tx740xNC356	D	6	0	54-64	60	2.62	11.66	0.02	1338837	3573312	GRMZM2G070500	nodulin MtN21 /EamA-like transporter family protein; auxin-induced protein 5NG4, putative, expressed	37898001
Tx740xNC356	I	6	30	64-65	65	2.30	10.48	0.02	94418426	95940965	GRMZM2G462760	SAUR-like auxin-responsive protein family ; OsSAUR25 - Auxin-responsive SAUR gene family member, expressed	2782579
Tx740xNC356	I	6	35	64-74	68	2.53	11.40	0.02	96558982	96880264	GRMZM2G462760	SAUR-like auxin-responsive protein family ; OsSAUR25 - Auxin-responsive SAUR gene family member, expressed	4322506
Tx740xNC356	D	6	67	64-85	84	3.20	14.11	0.02	141909772	143997309	dwil1	dwarf & irregular leaf1	346507
Tx740xNC356	I	6	75	42-62	56	2.50	9.25	0.02	154194879	154326722	GRMZM2G140805	nodulin MtN21 /EamA-like transporter family protein; auxin-induced protein 5NG4, putative, expressed	1385932

Supplemental Table S6. Continued.

Population	Trt †	Chr ‡	Pos (cM)	Sig. DAS§	Peak LOD¶ DAS††	LOD ¶	PVE #	Add (m) ††	Left marker (bp)	Right marker (bp)	Closest candidate gene	Gene function	Distance from gene (bp)
Tx740xNC356	I	6	76	51-62	62	2.28	9.12	0.02	154345260	155647497	GRMZM2G140805	nodulin MtN21 /EamA-like transporter family protein; auxin-induced protein 5NG4, putative, expressed	650354
Ki3xNC356	D	7	23	72-73	73	6.46	11.68	0.03	13980271	14905605	GRMZM2G320298	alpha/beta-Hydrolases superfamily protein; gibberellin receptor GID1L2, putative, expressed	7324200
Ki3xNC356	D	7	27	74-85	85	13.02	24.16	0.04	25781951	26153406	GRMZM2G075715	auxin response factor 6; auxin response factor, putative, expressed	13766771
Ki3xNC356	D	7	32	71-85	85	21.42	44.01	-0.05	95447274	96847624	GRMZM2G391795	alpha/beta-Hydrolases superfamily protein; gibberellin receptor GID1L2, putative, expressed	11778171
Ki3xNC356	D	8	3	64-85	85	3.58	6.08	-0.02	2792594	2901121	GRMZM2G000489	alpha/beta-Hydrolases superfamily protein; gibberellin receptor GID1L2, putative, expressed	12087236
LH82xLAMA	D	8	10	72-85	85	2.26	6.08	-0.02	5024449	5024479	GRMZM2G000489	alpha/beta-Hydrolases superfamily protein; gibberellin receptor GID1L2, putative, expressed	9909629
LH82xLAMA	D	8	84	35-43	43	3.08	8.40	0.01	157963615	157970345	GRMZM2G116557	auxin response factor 2; auxin response factor, putative, expressed	2134364
LH82xLAMA	D	8	96	51-61	54	4.26	11.35	0.03	165542954	165556999	GRMZM2G431066	SAUR-like auxin-responsive protein family ; OsSAUR24 - Auxin-responsive SAUR gene family member, expressed	349596
LH82xLAMA	D	8	97	44-50	50	3.97	10.62	0.02	166105809	166240043	GRMZM2G431066	SAUR-like auxin-responsive protein family ; OsSAUR24 - Auxin-responsive SAUR gene family member, expressed	972545
LH82xLAMA	D	9	14	24-34	24	2.23	5.71	0.00	5289590	6124392	GRMZM2G307440	alpha/beta-Hydrolases superfamily protein; gibberellin receptor GID1L2, putative, expressed	873137
LH82xLAMA	D	9	84	69-71	69	2.17	5.18	-0.02	139931280	139961881	GRMZM2G031447	carboxyesterase 17; gibberellin receptor GID1L2, putative, expressed	1592902
LH82xLAMA	I	9	94	61-62	62	2.28	5.68	-0.02	145282309	145505072	GRMZM2G028039 _gras45	GRAS family transcription factor; gibberellin response modulator protein, putative, expressed	3773603
LH82xLAMA	D	10	20	44-85	44	2.13	5.59	-0.01	5874629	6537612	cr4	Crinkly4; short plant	616214
LH82xLAMA	D	10	22	44-50	44	2.31	6.21	-0.01	5874629	6537612	cr4	Crinkly4; short plant	616214
LH82xLAMA	D	10	23	44-50	44	2.26	5.81	-0.01	5874629	6537612	cr4	Crinkly4; short plant	616214
LH82xLAMA	D	10	26	24-50	24	2.50	6.84	0.00	8451245	10196681	GRMZM2G346110	SAUR-like auxin-responsive protein family ; OsSAUR15 - Auxin-responsive SAUR gene family member	663550
LH82xLAMA	D	10	45	45-64	64	2.09	5.75	-0.02	82042041	82850420	GRMZM2G072632	Auxin efflux carrier family protein; auxin efflux carrier component, putative, expressed	7838586

Supplemental Table S6. Continued.

Population	Trt †	Chr ‡	Pos (cM)	Sig. DAS Int. §	Peak DAS #	Peak LOD ¶	Peak PVE ††	Peak Add ‡‡	Left marker (bp)	Right marker (bp)	Closest candidate gene	Gene function	Distance from gene (bp)
LH82xLAMA	I	10	46	51-60	58	4.72	10.43	-0.02	88270397	88401043	GRMZM2G072632	Auxin efflux carrier family protein; auxin efflux carrier component, putative, expressed	1949096
LH82xLAMA	I	10	47	61-62	62	4.65	12.75	-0.02	92823460	95666918	GRMZM2G072632	Auxin efflux carrier family protein; auxin efflux carrier component, putative, expressed	3960373
LH82xLAMA	D	10	48	62-66	62	2.60	7.07	-0.02	99931137	101933970	GRMZM2G137451	auxin signaling F-box 2; OsFBL16 - F-box domain and LRR containing protein, expressed	10066214
LH82xLAMA	D	10	51	33-66	34	2.54	7.08	0.00	127261630	127988227	GRMZM2G007481	Auxin efflux carrier family protein; auxin efflux carrier component, putative, expressed	828343
LH82xLAMA	D	10	57	23-85	27	2.55	6.96	0.00	135609157	136083608	GRMZM2G397684	brassinosteroid-responsive RING-H2; zinc finger, C3HC4 type domain containing protein, expressed	160978
LH82xLAMA	I	10	58	63-85	64	4.38	12.29	-0.03	136083608	136247247	GRMZM2G397684	brassinosteroid-responsive RING-H2; zinc finger, C3HC4 type domain containing protein, expressed	158068
LH82xLAMA	D	10	58	23-85	27	2.46	6.80	0.00	136083608	136247247	GRMZM2G397684	brassinosteroid-responsive RING-H2; zinc finger, C3HC4 type domain containing protein, expressed	158068
LH82xLAMA	D	10	65	20-22	20	2.65	7.22	0.00	140049011	140268347	GRMZM2G456644	SAUR-like auxin-responsive protein family ; OsSAUR20 - Auxin-responsive SAUR gene family member, expressed	1394099

† Trt: Treatment; I: Irrigated; D: Non-Irrigated.
‡ Chr: Chromosome.
§ Day interval where the QTL has a LOD>2 and PVE>3.
# Day which QTL has the greatest LOD score within the significant day interval.
¶ LOD: $-\log_{10}$(p-value).
†† PVE: Percent variance explained.
‡‡ Additive effect estimate (m)
