# Supplementary material for: Unoccupied aerial system enabled functional modeling of maize height reveals dynamic expression of loci
Source: Plant Direct. 2020 May 10;4(5):e00223. doi: 10.1002/pld3.223 (PMC7212003; doi:10.1002/pld3.223)
Supplement: Supplementary file 3 — File S1 [file PLD3-4-e00223-s003.pdf]

Supplemental File S1. Statistical methods to identify flight to remove from temporal dataset.

Qualitative, visual assessment of point clouds and image processing reports demonstrated reduced tie point matching leading to "black holes" in point clouds in later season flights (>70 DAS), with the eight occurring in the low altitude DJI flights. The higher altitude Tuffwing flights largely avoided these issues (1 flight). We hypothesize that the increased homogenous canopy appearance, coupled with reduced frame of reference of the low-altitude images led to the failure of the SfM algorithms to identify tie points for three-dimensional point cloud construction. These image processing results of inbred populations surveys were similar to image processing results of hybrid maize trial UAS surveys occurring in the previous season (Anderson et al., 2019), demonstrating that the level of inbreeding did not result in differential image processing quality. Following qualitative assessment for blunders and black holes, six DJI (35-72 DAS) and ten Tuffwing (64-133 DAS) flights (16 total) were identified as high-quality point clouds for extraction of height estimates.

It is known that the quality of information contained within UAS image derived datasets fluctuates by flight date and several statistical and qualitative approaches were used to further filter the temporal dataset. First, Tukey HSD mean comparisons were made for the P95 entry BLUPs, across flight dates for each population in each environment to identify flights that did not follow the standard sigmoidal growth pattern of maize (Archontoulis and Miguez, 2015; Anderson et al., 2019). Flights on 64, 68, and 71 DAS produced a canopy of plants (all entries) far shorter than expected, which significantly lowered means during the exponential phase of growth, so these were removed. Days 72 and 96 were removed due to significantly larger means in comparison to other dates past the curve's upper vertex within the period of terminal growth (>65 DAS), where height should be consistent. In theory, phenotypic variance of plant height should increase throughout the growing season and become consistent across terminal growth dates. Following this theory, we conducted an unequal variances test across flight dates and identified days 71, 83, 86, 96, 124, and 133 as dates which deviate from theory in two or more populations by environment grouping. Ignoring those flights with unequal variance resulted in eight high quality flight dates (35, 43, 57, 62, 65, 69, 100, and 117 DAS) used for the remainder of this study

References:

Anderson SL, Murray S, Malambo L, Ratcliff C, Popescu S, Cope D, Chang A, Jung J, Thomasson JA (2019) Prediction of maize grain yield before maturity using improved temporal height estimates of unmanned aerial systems. The Plant Phenome Journal **2**

Archontoulis SV, Miguez FE (2015) Nonlinear regression models and applications in agricultural research. Agronomy Journal **107:** 786-798
